# Anti-oxidative function of follicular fluid HDL and outcomes of modified natural cycle-IVF

**DOI:** 10.1038/s41598-019-49091-3

**Published:** 2019-09-06

**Authors:** Ruxandra A. Nagy, Aafke P. A. van Montfoort, Henk Groen, Irene Homminga, Daniela Andrei, Rima H. Mistry, Josephine L. C. Anderson, Annemieke Hoek, Uwe J. F. Tietge

**Affiliations:** 1 Department of Paediatrics, Centre for Liver, Digestive, and Metabolic Diseases, University of Groningen, University Medical Centre Groningen, 9713 GZ Groningen, The Netherlands; 2 Department of Obstetrics and Gynaecology, Section Reproductive Medicine, University of Groningen, University Medical Centre Groningen, 9713 GZ Groningen, The Netherlands; 3 Department of Obstetrics & Gynaecology, GROW School for Oncology and Developmental Biology, Maastricht University Medical Centre, 6229 HX Maastricht, The Netherlands; 4 Department of Epidemiology, University of Groningen, University Medical Centre Groningen, 9713 GZ Groningen, The Netherlands; 5 Division of Clinical Chemistry, Department of Laboratory Medicine, Karolinska Institutet, Stockholm, Sweden; 6 Clinical Chemistry, Karolinska University Laboratory, Karolinska University Hospital, SE-141 86 Stockholm, Sweden

**Keywords:** Predictive markers, Endocrine reproductive disorders

## Abstract

High density lipoproteins (HDL) are the main cholesterol carriers in follicular fluid (FF), the natural environment of oocyte development. Additionally, HDL have critical biological functions such as anti-oxidative capacity, which have not been studied in reproduction. Therefore, this study aimed to investigate whether the anti-oxidative function of FF-HDL is associated with fertility outcomes. From 253 women undergoing modified natural cycle (MNC)- IVF at a single academic centre FF and plasma were collected (n = 375 cycles). Anti-oxidative function of FF was mainly attributable to HDL (n = 8; 83%). FF-HDL had a higher anti-oxidative function than plasma HDL (n = 19, P < 0.001) coinciding with increased vitamin E and sphingosine 1 phosphate content (P = 0.028 each). Proteomic analysis indicated no significant differences in major anti-oxidative proteins such as paraoxonase 1, apolipoprotein (apo) A-I or apoA-IV between FF-HDL and matched plasma-HDL (n = 5), while apoC-III, apoE and apoC-II were relatively lower in FF-HDL. Finally, FF-HDL anti-oxidative function was related to a decrease in the odds of the oocyte undergoing normal fertilization, an association that persisted after adjustment for confounders (odds ratio 0.97 (0.93–1), P = 0.041). In conclusion, FF-HDL has considerable anti-oxidative properties that might be relevant for embryo quality.

## Introduction

The role of high density lipoproteins (HDL) in health and disease is traditionally viewed in the context of protection against atherosclerotic cardiovascular disease (CVD). Although circulating HDL-cholesterol levels are consistently inversely correlated with incident CVD events in large population studies, recent genetic research and disappointing pharmacological intervention studies resulted in a shift of concept towards HDL function being more important than HDL-cholesterol^1^. While the body of literature on HDL function in the cardiovascular field is increasing, this concept remains insufficiently explored in other fields where HDL might play a physiological role, such as reproduction^2^.

In female reproduction, follicular fluid (FF) is the natural environment for the final phase of oocyte maturation. Its composition has been linked to oocyte growth and quality, and variations in the metabolic profile of FF, such as lipoproteins, certain bile acid species, glucose, lactate and other metabolites are associated with the developmental potential of embryos in *in vitro* fertilization (IVF) procedures^3–5^. A key component of FF is cholesterol, which is almost exclusively contained within HDL, which are believed to originate from the blood compartment^3,6^. During follicular maturation, HDL cholesterol homeostasis in FF is important as it provides the substrate for local steroid hormone production^7^. Additionally, HDL quantity and structure has been linked to oocyte quality in mice as exemplified by the presence of dysfunctional oocytes and subsequent infertility in female mice lacking the HDL receptor SR-BI^8,9^. In addition to cholesterol transport, HDL have a number of functional properties. A key metric of HDL functionality is the ability to prevent oxidation^10^.

Oxidative stress has a negative impact on reproductive fitness as exemplified in women with reproductive disorders such as polycystic ovarian syndrome and endometriosis^11–14^. Furthermore, unfavourable metabolic conditions including obesity, insulin resistance or other components of the metabolic syndrome are associated with decreased fertility on the one hand and with altered plasma HDL anti-oxidative function on the other^10,15–19^. However, despite the biological activities of HDL and their abundant presence in FF, the anti-oxidative properties of HDL in FF and their potential association with parameters of fertility have not been explored thus far. Therefore, the present study aimed to evaluate the relevance of anti-oxidative properties of FF-HDL for outcomes of modified natural cycle (MNC)-IVF. In contrast to classic hyperstimulation IVF, in MNC-IVF (a setting close to normal reproductive physiology) it is possible to associate each FF sample collected with one specific oocyte and subsequently one embryo.

## Results

### Study population characteristics

**Figure 1:**
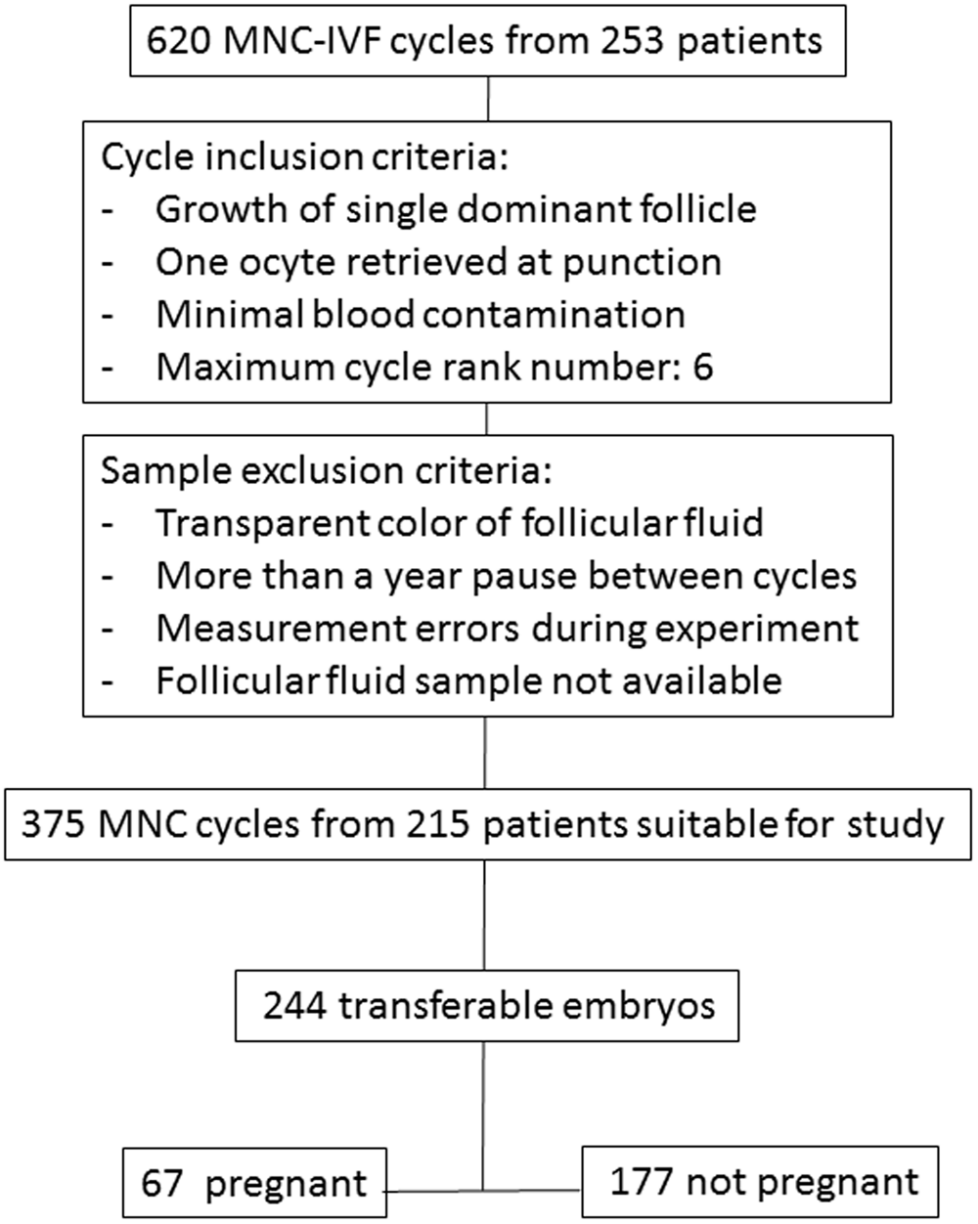
Flow diagram of sample selection. Of the 620 MNC-IVF cycles that were initially considered for inclusion in the study, 375 cycles were consistent with all of the selection criteria as detailed in methods. MNC, modified natural cycle.

As detailed in methods, FF and plasma were collected between August 2013 and April 2017 from a total of 620 MNC-IVF cycles derived from 253 patients (Fig. 1). Of these, 375 monofollicular MNC-IVF cycles corresponding to 215 patients were suitable for analysis. The vast majority of cycles were excluded because no oocyte was retrieved upon intended ovum pick-up or because there was visible blood contamination of FF. Out of the 375 cycles, 160 were obtained during the observational study period and 215 during the prospective study. In total, there were 244 transferable embryos that resulted in 67 pregnancies. Table 1 contains a description of HDL anti-oxidative capacity and patient characteristics at the time of ovum pick-up.

**Table 1:** HDL anti-oxidative capacity and cycle characteristics of patients undergoing modified natural cycle IVF.

	Total group	P value
Reduction in oxidation (%)	81.7 ± 6.8	—
Age (years)	31.3 ± 3.4	0.474
BMI (kg/m^2^)^a^	23.2 [21–26.2]	0.154
Smoking		0.266
Yes	40 (11%)	
No	195 (52%)	
Stopped before treatment	107 (28%)	
Unknown	33 (9%)	
Alcohol consumption		0.974
Yes	185 (49%)	
No	138 (37%)	
Unknown	52 (14%)	
Duration of subfertility (months)^b^	35.6 [23.7–50.1]	0.286
Indication		0.568
Male factor	261 (70%)	
Tubal factor	48 (13%)	
Unexplained	66 (17%)	
Fertility treatment		0.729
ICSI	309 (82%)	
IVF	66 (18%)	

P values are derived from generalized estimating equations and describe the effect of cycle characteristics on HDL anti-oxidative capacity.

Data are expressed as mean ± standard deviation, median [interquartile range] or number of cases (percentage) of outcome. The relationship between cycle characteristics of patients and reduction in oxidation was estimated by univariate GEE.

^a^BMI at start of the fertility treatment episode. Values are missing for 31 cycles corresponding to 22 patients.

^b^Duration of subfertility at intake. Data are missing for 14 cycles corresponding to 11 patients.

### Anti-oxidative capacity of follicular fluid HDL

First, we determined how the anti-oxidative capacity of FF (containing virtually exclusively HDL as the sole lipoprotein subclass) compares to that of plasma HDL (obtained by precipitation of apoB-containing lipoproteins). For 19 randomly selected patients who fulfilled the above-mentioned selection criteria and underwent a first cycle MNC-IVF, the anti-oxidative capacity of FF and matching apoB-depleted plasma was measured. As shown in the supplementary table there were no major differences in patient characteristics between the selected subgroup and the whole study population. The percentage reduction in oxidation was consistently significantly higher in FF as compared to plasma HDL (84% [79.2–87.1] versus 67.2% [63.8–70.1]; *p* < 0.001; Supplementary Fig.). The correlation between the anti-oxidative function of FF and that of matching plasma did not reach significance (r_s_ = 0.307, *p* = 0.201).

Next, we investigated the specific contribution of FF-HDL to the overall anti-oxidative properties of FF. For this purpose, eight patients were randomly selected who fulfilled the above-mentioned criteria and underwent a first cycle MNC-IVF. As indicated in the Supplementary Table, except for the lower proportion of patients who consumed alcohol, these patients did not differ in their clinical characteristics compared to the whole patient population. HDL was isolated from these FF samples by fast protein liquid chromatography (FPLC). Cholesterol measurements confirmed that almost all cholesterol in FF is contained within HDL particles^3^. Importantly, the anti-oxidative capacity of isolated FF-HDL accounted for 83% [77.2–90.4] of the total FF anti-oxidative capacity (whole FF 57.14% [55.15–58.89] versus FF-HDL 46.75% [45.35–50.75]).

**Figure 2:**
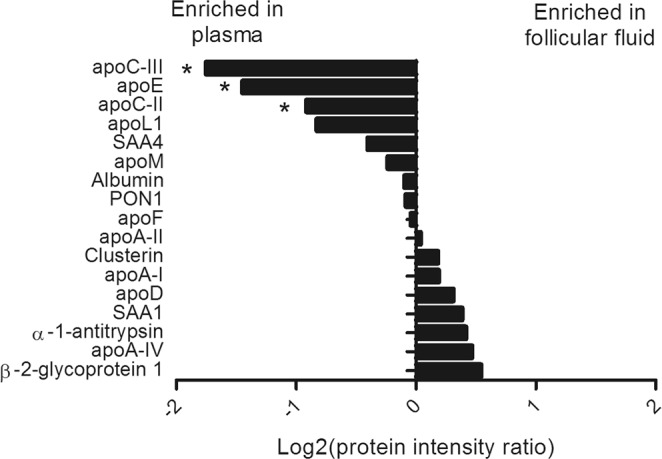
Comparison of the abundance of HDL-associated proteins between HDL from follicular fluid and from matched plasma (n = 6 each). Protein intensity ratio was calculated as the median protein intensity in follicular fluid-HDL samples divided by the median protein intensity in matched plasma-HDL samples. Apolipoprotein D and serum amyloid A1 could not be detected in plasma-HDL from one patient and in follicular fluid-HDL and plasma-HDL from three patients, respectively, hence the n number for these proteins is lower. *P < 0.05 by Wilcoxon Signed Ranks test (protein intensity). Apo – apolipoprotein, SAA – serum amyloid A, PON1 – paraoxonase/arylesterase 1.

In order to characterize the composition of FF-HDL in greater detail, proteomic analyses were carried out in matching plasma-HDL and FF-HDL (n = 6 patients) isolated by FPLC. FF-HDL and plasma-HDL from one patient with a high inflammatory load were subsequently excluded from the analysis. As summarized in Fig. 2 (for a complete list of proteins please see Supplementary Table 2) apolipoprotein C-III, apolipoprotein E and apolipoprotein C-II were significantly enriched in plasma-HDL as compared to FF-HDL (each P < 0.05). For the other proteins, notably including proteins implicated in oxidative stress defence such as PON1, apoA-I or apoA-IV, no significant differences between FF-HDL and matched plasma-HDL were discernible. Next, we explored the content of anti-oxidative lipids in the different HDL preparations. Vitamin E and sphingosine-1-phosphate (S1P) were both present in FF-HDL at significantly higher levels than in matched plasma-HDL (vitamin E: FF-HDL 4.21 [9.32–49.05] nmol/ml versus plasma-HDL 3.86 [2.50–27.41] nmol/ml, P = 0.028; S1P: FF-HDL 71.08 [36.83–340.72] ng/l versus plasma-HDL 34.60 [25.02–224.99] ng/l, P = 0.028).

### Intra-individual variation in the anti-oxidative function of follicular fluid HDL

The anti-oxidative function of FF-HDL was assessed in 215 patients (Fig. 1), and in 106 of them material from a minimum of two MNC-IVF cycles were available for comparison. For these, minimum and maximum values for FF-HDL anti-oxidant capacity were calculated and the difference was expressed as the intra-individual variance. There was a strong positive correlation between the minimum and maximum FF-HDL anti-oxidant function (minimum: 78.7 [75.5–81.6], maximum 85.1 [81–88], r_s_ = 0.508, *p* < 0.001, intra-individual variance 7.1 [3.3–11.4]), indicating that the anti-oxidative function of FF-HDL is a rather stable parameter.

### Follicular fluid HDL anti-oxidative function in relation to embryo quality and pregnancy

We investigated the relation between FF-HDL anti-oxidative function and embryo quality as well as pregnancy occurrence (Table 2). Of the total 309 cycles during which an ICSI procedure was planned, in seven cycles the procedure was not carried out due to separation of the oocyte from the zona pellucida or abnormal oocyte morphology. Of the remaining cycles, 16 oocytes were not injected due to insufficient spermatozoa. One oocyte was lost during removal of granulosa cells and after ICSI 15 oocytes degenerated. Of the total 366 oocytes (ICSI and IVF), in which development after fertilization was followed, 92 had an abnormal number of pronuclei or did not undergo cell cleavage and were therefore discarded. Finally, in 244 monofollicular MNC cycles the embryo was suitable for transfer, which resulted in 67 positive pregnancy tests from 64 patients.

**Table 2:** Oocyte and embryo development in Modified Natural Cycle IVF and ICSI and follicular fluid anti-oxidative capacity.

	Number of cycles	Anti-ox (%)
Progression to metaphase II (ICSI)	Yes (n = 287) No (n = 15)	81.7 ± 7.2 83.5 ± 6.3
Normal fertilization	Yes (n = 244) No (n = 92)	81.3 ± 6.8 82.8 ± 6.6
Fragmentation	Low (less than 10%) (n = 199) High (more than 10%) (n = 45)	81.6 ± 6.7 80.0 ± 6.9
Top quality embryo	Yes (n = 128) No (n = 208)	81.5 ± 6.9 81.9 ± 6.7
Pregnancy	Yes (n = 67) No (n = 177)	81.4 ± 7.4 81.3 ± 6.5

Data are expressed as mean ± standard deviation or median [interquartile range]. Anti-ox, anti-oxidative function.

A higher FF-HDL anti-oxidative capacity was associated with decreased odds of the oocyte undergoing normal fertilization (Table 3), an association that remained significant after adjustment for age, BMI and smoking (OR 0.97 [0.93–1.00], *p* = 0.041). No significant relationship with the corresponding oocyte (progression to metaphase II in ICSI), embryo (fragmentation, top quality embryo) and pregnancy parameters (positive pregnancy test) was found.

**Table 3:** Generalized estimating equations analysis of the relationship between embryo development in Modified Natural Cycle IVF and ICSI and follicular fluid anti-oxidative capacity.

	Unadjusted model		Adjusted model	
	Odds ratio (95% CI)	*p* value	Odds ratio (95% CI)	*p* value
Progression to metaphase II (ICSI)	0.97 (0.91–1.02)	0.220	0.97 (0.91–1.02)	0.225
Normal fertilization	**0**.**97** (**0**.**94**–**1**.**00)**	**0**.**042**	**0**.**97** (**0**.**93**–**1**.**00)**	**0**.**041**
Low fragmentation^a^	1.03 (0.98–1.08)	0.208	1.04 (1.00–1.09)	0.068
Top quality embryo	1.00 (0.97–1.03)	0.898	1.00 (0.97–1.04)	0.805
Pregnancy	1.00 (0.96–1.05)	0.906	1.01 (0.96–1.06)	0.703

Adjusted model includes percentage reduction in LDL oxidation, maternal age, BMI, smoking. Additionally, models for normal fertilization and pregnancy include the type of fertility treatment (IVF or ICSI) and duration of subfertility, respectively.

Bold values: *p* < 0.05 in GEE analysis.

^a^After exclusion of one extreme outlier (FF-HDL anti-oxidative function of 55.58%): adjusted OR 1.03 (0.98–1.08), *p* value = 0.202.

## Discussion

The results of this study demonstrate that (i) FF has anti-oxidative capacity of which the majority is attributable to the presence of HDL, and (ii) an increase in FF anti-oxidative function was associated with a decreased chance for the oocyte to undergo normal fertilization. The above findings suggest that beyond being a cholesterol carrier, FF-HDL have an anti-oxidative function that is related to the developmental potential of the oocyte during the initial phases of zygote development.

HDL are small lipoprotein particles of complex composition which are, according to our results, responsible for more than 80% of the total FF anti-oxidative function. Moreover, the significantly increased anti-oxidative properties of FF as compared to apolipoprotein B-depleted plasma indicate the existence of an environment relatively enriched in anti-oxidant capacity surrounding the developing oocyte. This finding is in accordance with previous work showing that FF from women undergoing (albeit hyperstimulation) IVF contains significantly lower levels of oxidative stress markers (conjugated dienes, lipid hydroperoxides and thiobarbituric acid reactive substances) and has a higher total antioxidant capacity as compared to plasma^20,21^. Extending current knowledge, the present study shows that the net pro-oxidative property of FF is mainly due to local HDL particles. Moreover, the anti-oxidative function of FF-HDL fluctuates within a narrow range with a relatively stable and low intra-individual variation between cycles.

Despite the expected diffusion of HDL from blood across the blood-follicle barrier, FF and plasma-derived HDL differ in size and composition, indicating that during transport, remodelling of HDL occurs^6^. In support of such a concept, we detected significant differences in the HDL proteome between the two matrices, namely a decreased apolipoprotein E, apolipoprotein C-II and apolipoprotein C-III content of FF-HDL. However, more obvious candidates for HDL-associated proteins with an anti-oxidative function such as PON1, apolipoprotein A-I, apolipoprotein A-IV or the S1P carrier apolipoprotein M were not differentially present on FF compared with plasma HDL. In contrast, FF-HDL was enriched in vitamin E and S1P, which may conceivably contribute to the accentuated anti-oxidative potential of FF as compared to plasma HDL. Previous literature reported lower levels of apolipoprotein A-I and apolipoprotein A-IV in apolipoprotein B-precipitated FF-HDL than in matched serum or plasma^6,22^. This discrepancy is likely due to different stimulation protocols; specifically, the higher amounts of hormones used in hyperstimulation-IVF in previous literature may influence oxidative balance in the follicular environment.

The present study found a negative correlation between the FF-HDL anti-oxidative capacity and the chance of the oocyte undergoing normal fertilization, which may seem counterintuitive. However, the optimum balance of pro- and anti-oxidants for successful fertilization has yet to be established, and previous literature implies that both extremes of the oxidative stress spectrum may be detrimental for oocyte maturation and embryo quality. Specifically, our finding is in agreement with the study of Sabatini *et al*. who reported an inverse correlation between FF levels of the antioxidant superoxide dismutase and fertilization rates^23^. Moreover, anti-oxidative defence may increase in response to high stress, such as seen for example in smokers^24^. Increased anti-oxidant function may thus reflect the need of the oocyte for increased protection in unfavourable developmental conditions and is likely part of a functioning defence mechanism against oxidative stress. Further, a certain amount of oxidative stress may be necessary for normal reproductive physiology, as is suggested by a previous study in which all pregnancies occurred in women with mid-range FF oxidative-stress indices^25^. In addition, oxygen radicals represent an ancient signalling system important for many biological processes, among others for providing guidance cues during development^26^. On the other hand, Oyawoye *et al*. found that an increase in the total antioxidant capacity of FF was associated with successful oocyte fertilization, which may reflect the detrimental effects of excess oxidative stress^27^. Efforts have also been made to determine the upper limit of ROS beyond which it becomes toxic for the oocyte^28^. However, it is rather difficult to draw an exact conclusion about the physiological limits for the pro-/anti-oxidant balance due to different laboratory pro-/anti-oxidant measurement techniques and hormonal stimulation protocols used in fertility research.

The present study did not find a relationship between the FF-HDL anti-oxidative function and the occurrence of pregnancy, which is in agreement with previous work^20^. This finding may be attributable to the fact that, aside from the pre-ovulatory environment of oocyte development, extra-ovarian factors such as endometrial receptivity and sperm quality are crucial determinants of pregnancy. Nonetheless, previous studies have reached conflicting conclusions about a possible detrimental versus beneficial role of FF oxidative stress on the chances of achieving a successful pregnancy^21,29–32^. The difference in study outcomes may be explained by differences in IVF procedures and patient characteristics. In MNC-IVF, the patient’s natural ovulatory cycles are followed, hence a much lower dose of gonadotropins is administered which is likely to influence the FF total antioxidant capacity^33^. The lower doses of exogenous hormones result in the development of only one dominant follicle. Consequently, only one oocyte is fertilized and single embryo transfer is performed. MNC-IVF is thus much closer to normal physiology of ovarian follicle growth as compared to the conventional hyperstimulation protocols.

We therefore consider one of the strengths of our study the use of material from MNC-IVF, which allows for correlating components of FF with oocyte, embryo and pregnancy outcomes. Additionally, the current study highlights one of the previously unrecognized key players in the FF oxidative balance, namely HDL, and quantified its contribution to the total local functional antioxidant capacity. The present study also has certain limitations. Firstly, this is a single centre study. Secondly, dynamic HDL function assays are not standardized as e.g. clinical chemistry determinations of static biomarkers, and results have to be interpreted in the context of the respective conditions applied^10^. Lastly, due to the invasive nature of the oocyte pick-up procedure, minor contamination of the FF samples with blood cannot be completely excluded. However, since the anti-oxidative capacity of FF-HDL is higher, that would rather be expected to result in a dilution of the observed effect size.

Investigating HDL function is an emerging concept in cardiovascular medicine, and several studies have found an increased prevalence of cardiovascular disease risk in women with ovulatory failure due to e.g. polycystic ovarian disease, endometriosis or primary ovarian insufficiency^16–18^. Given the perceived ability of HDL to diffuse across the ovarian blood-follicle barrier, lifestyle and drug interventions to modify the functionality of HDL may also affect reproductive outcomes by e.g. interfering with known risk factors for infertility such as the components of the metabolic syndrome in which HDL dysfunction may be expected^34–38^.

In summary, this study shows that FF is a matrix with accentuated anti-oxidative properties that are predominantly exerted by HDL in the pre-ovulatory follicular environment. Moreover, differences in anti-oxidative capacity between FF- and plasma-HDL might be explained by an altered lipid composition of the particles. Importantly, an increase in the anti-oxidative function of FF-HDL seems to be a negative predictor of normal fertilization, which may be the reflection of an unfavourable ovarian environment that stimulated anti-oxidant activity. Further studies are warranted to gain more insight into the relationship between FF-HDL function and embryo quality as well as pregnancy chances in IVF. Moreover, fine-tuning the balance between oxidative stress and antioxidants is conceivably required in order to exploit this system for therapeutic purposes, with the ultimate goal of optimizing reproductive success in IVF procedures.

## Methods

### Sample and data collection

Surplus FF and blood from ovulatory women attending the fertility clinic of the University Medical Centre Groningen in the years 2013–2017 for a maximum of six cycles of MNC-IVF was used for the current study. Patients attending the IVF clinic between August 2013 and July 2014 were included in an observational setting as part of routine medical care. From October 2014 to April 2017 IVF patients were enrolled in a prospective observational cohort study on the relationship between women’s nutrition, blood biomarkers, FF composition and MNC-IVF outcome parameters. There were no differences in hormonal treatment, ovum pick-up or laboratory procedures between the two sequentially recruited patient groups.

On the day of oocyte retrieval, a fasted blood sample was drawn and the surplus plasma was stored at −80 °C. After the oocyte had been collected for IVF/ICSI, the FF was centrifuged for 20 minutes at 300 g and the supernatant was stored at −80 °C for later analysis. FF supernatant with visible or suspected blood contamination was discarded. Materials from each cycle were assigned non-traceable codes and information regarding the participants and IVF outcomes were gathered from a database for routine patient care. MNC-IVF has the advantage of being as close to normal reproductive physiology as possible.

For the present study, samples were used from cycles in which (i) one dominant follicle developed, (ii) one oocyte was retrieved and (iii) macroscopic blood contamination of FF was minimal.

### Ethical approval

For the present study, plasma and FF were used, which are considered waste materials that would otherwise be discarded. For the prospective study (Netherlands Trial Register number NTR4409), patients were asked to fill in food frequency questionnaires; for this additional task, medical ethics committee approval was obtained (Medisch Ethische Toetsingscommissie 2014/007, number NL47569.042.13) and all participants gave written informed consent before inclusion in the study. For the usage of samples collected during routine medical care, approval was requested at the Medical Ethical Committee of the UMCG but waived, since all patients had signed a universal consent form that permits the usage of surplus material and data from medical records for research purposes under the condition that the patient identity remains untraceable and the data confidential. The research was conducted in accordance with the Declaration of Helsinki.

### Fertility treatment

The MNC-IVF procedure was carried out as previously described (Supplementary methods)^5^. The oocyte underwent standard insemination in the form of incubation with medium containing spermatozoa or intracytoplasmatic sperm injection (ICSI) within 6 h of retrieval. The number of pronuclei was assessed on the morning of day 1 after insemination/spermatozoa injection and cleavage, the number of blastomeres, percentage of fragmentation and presence or absence of multinucleated blastomeres (MNBs) on day 2 after insemination. Normal fertilization was defined as the presence of either zero, one or two pronuclei on day 1 and the occurrence of cell cleavage on day two. Abnormal fertilization was defined as the presence of three pronuclei on day 1 or the absence of cell cleavage on day two, irrespective of the number of pronuclei on day 1. Top quality embryos were defined as the presence of two pronuclei on day 1 and presence of four cells, absence of MNBs and less than 20% fragmentation on day two after fertilization. Single embryo transfer took place on day two after oocyte retrieval. Embryos containing more than two pronuclei or more than 40% fragmentation were discarded. On days 5, 8 and 11 after oocyte retrieval, hCG (1500 IU, Pregnyl, MSD) was administered for luteal support. The occurrence of pregnancy was defined by a positive serum hCG test at two weeks after embryo transfer. Ongoing pregnancy (defined as the presence of an intrauterine gestational sac with fetal heartbeat) was confirmed by ultrasound at 12 weeks gestational age.

### HDL anti-oxidative function

HDL was isolated from plasma by precipitating apoB-containing lipoproteins using polyethylene glycol-6000 (Sigma-Aldrich, Germany) exactly as detailed previously^39^. For FF samples, apoB-containing lipoproteins were not precipitated, since FF is already almost entirely devoid of these^6^.

The anti-oxidative capacity of FF and plasma HDL was studied by adding individual FF and apoB-depleted plasma samples to native, unoxidized LDL particles as previously published^39–43^. Oxidation was induced by 2,2’-azobis [2-amidinopropane] dihydrochloride (2.5 mM; AAPH, Sigma, Germany) in aliquots of native LDL (3.6 mM cholesterol concentration), to which either PBS (2.5 µl; control) or FF (2.5 µl) or apoB-depleted plasma (2.5 µl) was added. Anti-oxidative function was calculated as the capacity of HDL to suppress the maximally induced LDL oxidation. Consequently, higher values describe lower oxidation, and thus better protection. The inter-assay coefficient of variation of the assay was previously determined to be 5.1%^19,40,42,43^.

To assess the contribution of FF-HDL to the total FF anti-oxidative capacity, HDL was isolated from eight first cycle MNC FF samples from individual patients by fast protein liquid chromatography (FPLC)^44^. Cholesterol content in individual fractions after FPLC was measured using a routine enzymatic colorimetric method (Roche Diagnostics GmbH, The Netherlands). For each sample, the FPLC fractions corresponding to HDL (as determined by fraction number and cholesterol content) were pooled and anti-oxidant capacity was subsequently determined as described above in the concentrated HDL and in the whole FF sample (in total 16 samples corresponding to the above-mentioned eight patients).

### HDL composition

Differential protein composition of plasma- versus FF-HDL particles isolated by FPLC was assessed by untargeted proteomics as follows. First, for in solution digestion 10 µL of 0.2% Rapigest in milliQ water was added to the protein mixtures (50 µg) followed by 10 µL of 100 mM Ammonium BiCarbonate (ABC) in milliQ water. The sample was reduced by adding 10 µL of 10 mM DTT in 100 mM ABC buffer and incubated at 56 °C for 30 minutes. The sample was cooled down to room temperature (RT) and alkylated by adding 10 µL of a 55 mM Iodoacetamide in 100 mM ABC buffer and left at RT in the dark for 45 minutes. Alkylation was quenched by addition of 10 µL 10 mM DTT in 100 mM ABC buffer. 25 µL of a 10 ng/µl trypsin in 100 mM ABC buffer was added and the sample was incubated in an oven at 37 °C. After two hours an additional 25 µL of a 10 ng/µl trypsin in 100 mM ABC buffer was added and incubated overnight at 37 °C. Samples were acidified by addition of 10 µL 10% (v/v) TFA in water and incubated at 37 °C. After 45 minutes the samples were spun down and the supernatant was concentrated and desalted by Stage Tip cleanup and finally eluted in 20 µL 50% acetonitrile/0.1%TFA. The samples were dried in a speedvac and resuspended in 20 µl 5% formic acid for analysis by LCMS. LC-MS/MS of the tryptic peptides was performed with the Ultimate 3000 HPLC system coupled online to a Q-Exactive-Plus mass spectrometer with a NanoFlex source (both from Thermo Fisher Scientific) equipped with a stainless-steel emitter. 1 µL of tryptic digest was loaded onto a 5 mm × 300 µm i.d. trapping micro column packed with PepMAP100 5 µm particles (Dionex) in 0.1% FA at the flow rate of 20 µL/min. After loading and washing for 3 minutes, peptides were back-flush eluted onto a 50 cm × 75 µm i.d. nano-column, packed with Acclaim C18 PepMAP100 2 µm particles (Dionex). The following mobile phase gradient was delivered at the flow rate of 300 nL/min: 2–45% of solvent B in 90 min; 50–80% B in 1 min; 80% B during 14 min, and back to 3% B in 1 min and held at 2% B for 24 minutes. Solvent A was 100:0 H_2_O/acetonitrile (v/v) with 0.1% formic acid and solvent B was 0:100 H_2_O/acetonitrile (v/v) with 0.1% formic acid. The mass spectrometer was operated in data-dependent mode to switch automatically between MS and MS/MS and the top 15 precursor ions with a charge state of 2–5+ were selected with an isolation width of 1.8 m/z and fragmented by HCD (resolution 17500, max IT = 50 ms) with a 20 seconds exclusion time. The normalized collision energy was set at 28. For database search and label free quantitation (LFQ) the software PEAKS X (Bioinformatics Solutions Inc., Waterloo, Ontario, Canada) was applied to the spectra generated by the Q-exactive plus mass spectrometer to search against the Human SwissProt database (2019.04.02/20404 entries) with fixed modification carbamidomethylation of cysteine and the variable post translational modifications oxidation of methionine (done with a maximum of 5 posttranslational modifications per peptide at a parent mass error tolerance of 20 ppm and a fragment mass tolerance of 0.04 Da). False discovery rate was set at 1 and at least 1 unique peptide should be present. LFQ was performed using the PeaksQ module incorporated in the Peaks X software.

Vitamin E and sphingosine-1-phosphate (S1P) were measured in HDL isolated from FF and plasma by FPLC using commercially available ELISA kits (MBS163944 and MBS163661, MyBioSource, San Diego, USA) following the instructions provided by the manufacturer. Final volume after pooling of FPLC HDL fractions and concentration of the sample was used to normalize the results.

### Statistical analysis

Results are expressed as mean ± standard deviation for normally distributed variables and median [interquartile range] for non-normally distributed variables. Extreme outliers were defined as cases with values more than three times the interquartile range above the third quartile or below the first quartile. Statistical analysis after exclusion of extreme outliers was compared with original results. Differences in HDL anti-oxidative capacity between FF and matched plasma, between the maximum and minimum HDL anti-oxidative function and between lipid and protein content of FF-HDL and plasma-HDL were analysed by means of Wilcoxon Signed Ranks test (paired), and their correlation was expressed as Spearman’s r. To study the relationship between FF anti-oxidative function and oocyte, embryo and pregnancy parameters, as well as the relationship between FF anti-oxidative function and cycle characteristics, multilevel analysis using generalized estimating equations (GEE) was used^45^. The results of GEE analysis are presented as odds ratio (95% confidence interval). A *p* value lower than 0.05 was considered statistically significant. Factors known from literature to influence fertility (age, BMI, smoking, type of procedure – IVF or ICSI – specifically for fertilization) and factors that were significantly correlated to MNC-IVF outcomes were included in the adjusted GEE model. SPSS 23 (SPSS, Inc., Chicago, IL, USA) was used for data analysis.

## Supplementary information

Supplementary methods, figure and tables

## Data Availability

The datasets generated during and/or analyzed during the current study are available from the corresponding author on reasonable request.
