# Supplementary material for: Anti-oxidative function of follicular fluid HDL and outcomes of modified natural cycle-IVF
Source: Sci Rep. 2019 Sep 6;9:12817. doi: 10.1038/s41598-019-49091-3 (PMC6731220; doi:10.1038/s41598-019-49091-3)
Supplement: Supplementary file 1 — Supplementary methods, figure and tables [file 41598_2019_49091_MOESM1_ESM.pdf]

Anti-oxidative function of follicular fluid HDL and outcomes of modified natural cycle-IVF

Ruxandra A. Nagy[a,b] , Aafke P.A. van Montfoort[b,c], Henk Groen[d], Irene Homminga[2], Daniela Andrei[a,b], Rima H. Mistry[a], Josephine L.C. Anderson[a], Annemieke Hoek[b], & Uwe J.F. Tietge[a,e,f,*]

[a] Department of Pediatrics, Center for Liver, Digestive, and Metabolic Diseases, University of Groningen, University Medical Center Groningen, 9713 GZ Groningen, The Netherlands

[b] Department of Obstetrics and Gynaecology, Section Reproductive Medicine University of Groningen, University Medical Centre Groningen, 9713 GZ Groningen, The Netherlands

[c] Department of Obstetrics & Gynaecology, GROW School for Oncology and Developmental Biology, Maastricht University Medical Centre, 6229 HX Maastricht, The Netherlands

[d] Department of Epidemiology, University of Groningen, University Medical Center Groningen, 9713 GZ Groningen, The Netherlands

[e] Division of Clinical Chemistry, Department of Laboratory Medicine, Karolinska Institutet, Stockholm, Sweden

[f] Clinical Chemistry, Karolinska University Laboratory, Karolinska University Hospital, SE-141 86 Stockholm, Sweden

*****Corresponding author: Dr. Uwe Tietge;

Division of Clinical Chemistry, Department of Laboratory Medicine (LABMED), H5, Alfred Nobels Alle 8, Karolinska Institutet. S- 141 83 Stockholm, Sweden.

Phone: +46852483723; email: uwe.tietge@ki.se or u_tietge@yahoo.com

Supplementary methods

Follicular growth was followed by vaginal ultrasound and serum estradiol and luteinizing hormone levels were measured regularly beginning at cycle days 6–8. When the diameter of the follicle reached 14 mm, the patient was started on daily injections of 0.25 mg GnRH antagonist cetrorelix (Cetrotide®, Merck bv, The Netherlands) and 150 IU recombinant FSH (r-FSH, Follitropin-alpha: Gonal-F®, Merck bv, The Netherlands). Patients were instructed to self-administer the subcutaneous injections at 24 hour intervals, with cetrorelix being used up to and including the day of ovulation triggering and r-FSH up to the day of ovulation triggering.

In order to induce ovulation, 10 000 IU hCG (Pregnyl®, Organon, The Netherlands) was administered when the dominant follicle reached a minimal size of 18 mm and/or serum estradiol levels exceeded 0.8 nmol/l. Approximately 34 hours after hCG administration oocyte retrieval was carried out by ultrasound-guided transvaginal follicle aspiration without sedation or local anesthesia, with a single-lumen aspiration needle and without flushing of the follicle.

Supplementary figure. Comparison of HDL anti-oxidative function in apoB-depleted plasma and matched follicular fluid (N=19). The anti-oxidative function of follicular fluid was significantly higher than that of matching apoB-depleted plasma. This indicates that follicular fluid is a compartment with augmented anti-oxidative properties. *** - P<0.001; anti-ox, anti-oxidative.

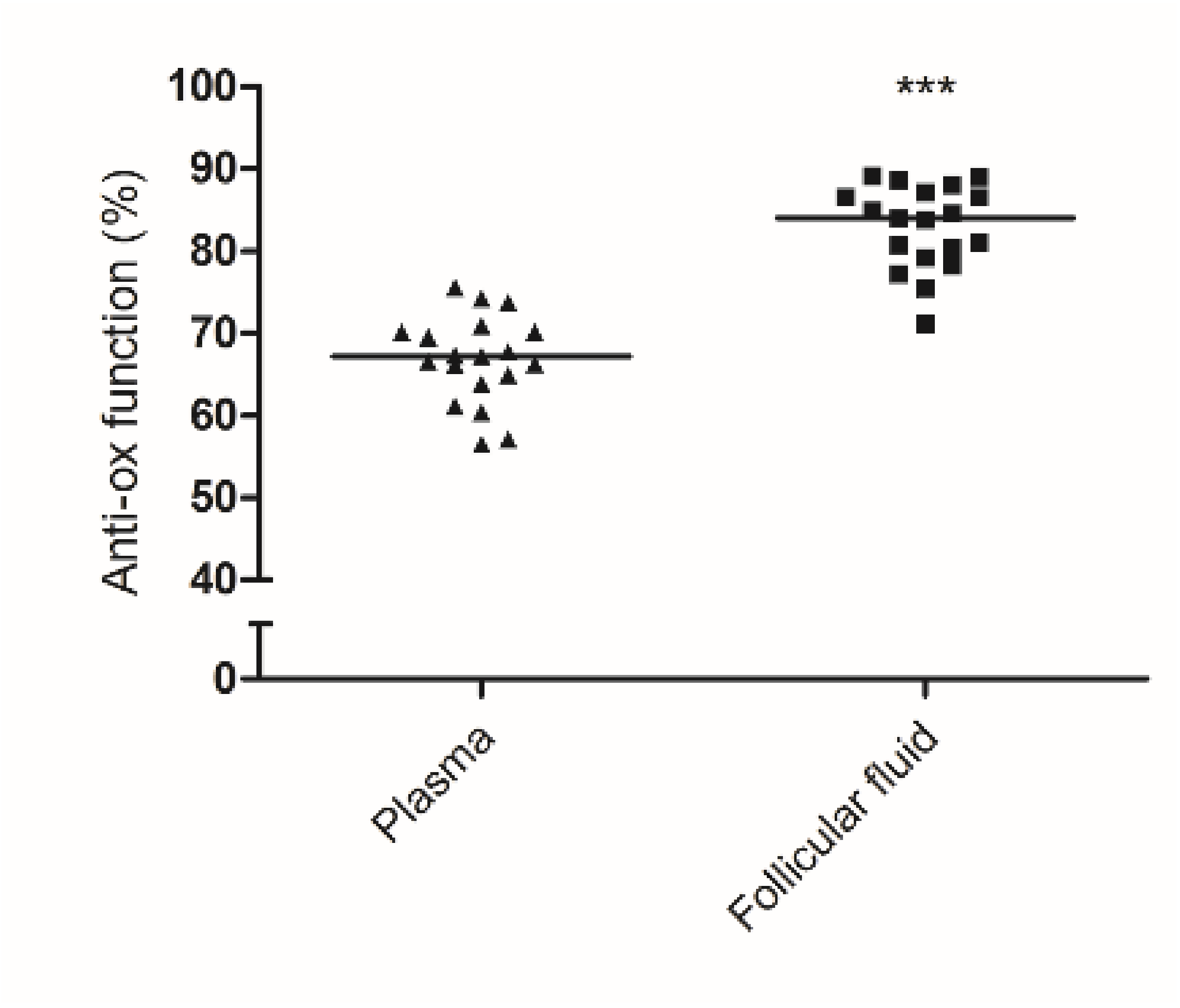

Supplementary table 1. Comparison of cycle characteristics of subgroups of patients for the comparison of FF versus matched plasma as well as total FF versus FF-HDL subfractions.

	Total group (N = 375)	Subgroup for comparison of FF and matched plasma (N = 19)	Subgroup for comparison of FF and HDL fraction (N = 8)
Age (years)	31.7 [29.3-33.7]	33.9 [29.9-34.9]	31.6 [28.9-33.4]
BMI (kg/m^2)[a]	23.2 [21.0-26.2]	25.8 [21.1-26.5]	23.2 [20.3-24.2]
Smoking			
Yes	40 (11%)	3 (16%)	0 (0%)
No	195 (52%)	6 (47%)	5 (63%)
Stopped before treatment	107 (28%)	6 (32%)	3 (37%)
Unknown	33 (9%)	1 (5%)	0 (0%)
Alcohol consumption			
Yes	185 (49%)	11 (58%)	3 (38%)
No	138 (37%)	7 (37%)	5 (63%)
Unknown	52 (14%)	1 (5%)	0 (0%)
Duration of subfertility (months)[b]	35.6 [23.7-50.1]	35.5 [18.6-43.8]	35.6 [20.1-38.4]
Indication			
Male factor	261 (70%)	14 (74%)	7 (88%)
Tubal factor	48 (13%)	3 (16%)	1 (12%)
Unexplained	66 (17%)	2 (10%)	0 (0%)
Fertility treatment			
ICSI	309 (82%)	16 (84%)	7 (88%)
IVF	66 (18%)	3 (16%)	1 (12%)

Supplementary table 2. Overview of proteins detected by untargeted proteomics in HDL from follicular fluid and matched plasma.

Accession	Description
P35908|K22E_HUMAN	Keratin type II cytoskeletal 2 epidermal OS=Homo sapiens OX=9606 GN=KRT2 PE=1 SV=2
P13645|K1C10_HUMAN	Keratin type I cytoskeletal 10 OS=Homo sapiens OX=9606 GN=KRT10 PE=1 SV=6
#CONTAM#P13645	SWISS-PROT:#CONTAM#P13645 Tax_Id=9606 Gene_Symbol=KRT10 Keratin type I cytoskeletal 10
P04264|K2C1_HUMAN	Keratin type II cytoskeletal 1 OS=Homo sapiens OX=9606 GN=KRT1 PE=1 SV=6
P81605|DCD_HUMAN	Dermcidin OS=Homo sapiens OX=9606 GN=DCD PE=1 SV=2
Q96KN2|CNDP1_HUMAN	Beta-Ala-His dipeptidase OS=Homo sapiens OX=9606 GN=CNDP1 PE=1 SV=4
#CONTAM#P35527	SWISS-PROT:#CONTAM#P35527 Tax_Id=9606 Gene_Symbol=KRT9 Keratin type I cytoskeletal 9
P35527|K1C9_HUMAN	Keratin type I cytoskeletal 9 OS=Homo sapiens OX=9606 GN=KRT9 PE=1 SV=3
P01767|HV353_HUMAN	Immunoglobulin heavy variable 3-53 OS=Homo sapiens OX=9606 GN=IGHV3-53 PE=1 SV=2
A3KN83|SBNO1_HUMAN	Protein strawberry notch homolog 1 OS=Homo sapiens OX=9606 GN=SBNO1 PE=1 SV=1
A6NCI4|VWA3A_HUMAN	von Willebrand factor A domain-containing protein 3A OS=Homo sapiens OX=9606 GN=VWA3A PE=2 SV=3
Q9H497|TOR3A_HUMAN	Torsin-3A OS=Homo sapiens OX=9606 GN=TOR3A PE=1 SV=1
P02751|FINC_HUMAN	Fibronectin OS=Homo sapiens OX=9606 GN=FN1 PE=1 SV=4
Q9BSJ2|GCP2_HUMAN	Gamma-tubulin complex component 2 OS=Homo sapiens OX=9606 GN=TUBGCP2 PE=1 SV=2
Q9Y230|RUVB2_HUMAN	RuvB-like 2 OS=Homo sapiens OX=9606 GN=RUVBL2 PE=1 SV=3
Q9Y4D8|HECD4_HUMAN	Probable E3 ubiquitin-protein ligase HECTD4 OS=Homo sapiens OX=9606 GN=HECTD4 PE=1 SV=5
P13598|ICAM2_HUMAN	Intercellular adhesion molecule 2 OS=Homo sapiens OX=9606 GN=ICAM2 PE=1 SV=2
P06312|KV401_HUMAN	Immunoglobulin kappa variable 4-1 OS=Homo sapiens OX=9606 GN=IGKV4-1 PE=1 SV=1
Q7Z494|NPHP3_HUMAN	Nephrocystin-3 OS=Homo sapiens OX=9606 GN=NPHP3 PE=1 SV=1
Q03591|FHR1_HUMAN	Complement factor H-related protein 1 OS=Homo sapiens OX=9606 GN=CFHR1 PE=1 SV=2
#CONTAM#P02769	SWISS-PROT:#CONTAM#P02769 (Bos taurus) Bovine serum albumin precursor
P05156|CFAI_HUMAN	Complement factor I OS=Homo sapiens OX=9606 GN=CFI PE=1 SV=2
O00391|QSOX1_HUMAN	Sulfhydryl oxidase 1 OS=Homo sapiens OX=9606 GN=QSOX1 PE=1 SV=3
P19652|A1AG2_HUMAN	Alpha-1-acid glycoprotein 2 OS=Homo sapiens OX=9606 GN=ORM2 PE=1 SV=2
Q6T4P5|PLPR3_HUMAN	Phospholipid phosphatase-related protein type 3 OS=Homo sapiens OX=9606 GN=PLPPR3 PE=2 SV=1
P04406|G3P_HUMAN	Glyceraldehyde-3-phosphate dehydrogenase OS=Homo sapiens OX=9606 GN=GAPDH PE=1 SV=3
P02763|A1AG1_HUMAN	Alpha-1-acid glycoprotein 1 OS=Homo sapiens OX=9606 GN=ORM1 PE=1 SV=1
P26927|HGFL_HUMAN	Hepatocyte growth factor-like protein OS=Homo sapiens OX=9606 GN=MST1 PE=1 SV=2
P08603|CFAH_HUMAN	Complement factor H OS=Homo sapiens OX=9606 GN=CFH PE=1 SV=4
A0A0C4DH34|HV428_HUMAN	Immunoglobulin heavy variable 4-28 OS=Homo sapiens OX=9606 GN=IGHV4-28 PE=3 SV=1

85	A0A0B4J1V6|HV373_HUMAN	Immunoglobulin heavy variable 3-73 OS=Homo sapiens OX=9606 GN=IGHV3-73 PE=3 SV=1
86	O60347|TBC12_HUMAN	TBC1 domain family member 12 OS=Homo sapiens OX=9606 GN=TBC1D12 PE=1 SV=3
87	P01344|IGF2_HUMAN	Insulin-like growth factor II OS=Homo sapiens OX=9606 GN=IGF2 PE=1 SV=1
88	Q5T7N2|LITD1_HUMAN	LINE-1 type transposase domain-containing protein 1 OS=Homo sapiens OX=9606 GN=L1TD1 PE=1 SV=1
89	P06681|CO2_HUMAN	Complement C2 OS=Homo sapiens OX=9606 GN=C2 PE=1 SV=2
90	Q9UNH5|CC14A_HUMAN	Dual specificity protein phosphatase CDC14A OS=Homo sapiens OX=9606 GN=CDC14A PE=1 SV=1
91	P02774|VTDB_HUMAN	Vitamin D-binding protein OS=Homo sapiens OX=9606 GN=GC PE=1 SV=2
92	Q13615|MTMR3_HUMAN	Myotubularin-related protein 3 OS=Homo sapiens OX=9606 GN=MTMR3 PE=1 SV=3
93	P01008|ANT3_HUMAN	Antithrombin-III OS=Homo sapiens OX=9606 GN=SERPINC1 PE=1 SV=1
94	Q6Y7W6|GGYF2_HUMAN	GRB10-interacting GYF protein 2 OS=Homo sapiens OX=9606 GN=GIGYF2 PE=1 SV=1
95	P01701|LV151_HUMAN	Immunoglobulin lambda variable 1-51 OS=Homo sapiens OX=9606 GN=IGLV1-51 PE=1 SV=2
96	P02750|A2GL_HUMAN	Leucine-rich alpha-2-glycoprotein OS=Homo sapiens OX=9606 GN=LRG1 PE=1 SV=2
97	A0A087WSY6|KVD15_HUMAN	Immunoglobulin kappa variable 3D-15 OS=Homo sapiens OX=9606 GN=IGKV3D-15 PE=3 SV=6
98	Q92820|GGH_HUMAN	Gamma-glutamyl hydrolase OS=Homo sapiens OX=9606 GN=GGH PE=1 SV=2
99	Q99996|AKAP9_HUMAN	A-kinase anchor protein 9 OS=Homo sapiens OX=9606 GN=AKAP9 PE=1 SV=4
100	Q8NFU7|TET1_HUMAN	Methylcytosine dioxygenase TET1 OS=Homo sapiens OX=9606 GN=TET1 PE=1 SV=2
101	O15047|SET1A_HUMAN	Histone-lysine N-methyltransferase SETD1A OS=Homo sapiens OX=9606 GN=SETD1A PE=1 SV=3
102	#CONTAM#Q9TRI1	TREMBL:#CONTAM#Q9TRI1 (Bos taurus) similar to inter-alpha-trypsin inhibitor heavy chain2
103	Q7Z388|D19L4_HUMAN	Probable C-mannosyltransferase DPY19L4 OS=Homo sapiens OX=9606 GN=DPY19L4 PE=1 SV=1
104	P27694|RFA1_HUMAN	Replication protein A 70 kDa DNA-binding subunit OS=Homo sapiens OX=9606 GN=RPA1 PE=1 SV=2
105	Q9Y666|S12A7_HUMAN	Solute carrier family 12 member 7 OS=Homo sapiens OX=9606 GN=SLC12A7 PE=1 SV=3
106	P36980|FHR2_HUMAN	Complement factor H-related protein 2 OS=Homo sapiens OX=9606 GN=CFHR2 PE=1 SV=1
107	P01743|HV146_HUMAN	Immunoglobulin heavy variable 1-46 OS=Homo sapiens OX=9606 GN=IGHV1-46 PE=1 SV=2
108	P02765|FETUA_HUMAN	Alpha-2-HS-glycoprotein OS=Homo sapiens OX=9606 GN=AHSG PE=1 SV=2
109	P01042|KNG1_HUMAN	Kininogen-1 OS=Homo sapiens OX=9606 GN=KNG1 PE=1 SV=2
110	P29622|KAIN_HUMAN	Kallistatin OS=Homo sapiens OX=9606 GN=SERPINA4 PE=1 SV=3
111	P02749|APOH_HUMAN	Beta-2-glycoprotein 1 OS=Homo sapiens OX=9606 GN=APOH PE=1 SV=3
112	Q6ZRG5|YQ015_HUMAN	Putative uncharacterized protein FLJ43944 OS=Homo sapiens OX=9606 PE=5 SV=1
113	P02533|K1C14_HUMAN	Keratin type I cytoskeletal 14 OS=Homo sapiens OX=9606 GN=KRT14 PE=1 SV=4
114	#CONTAM#P02533	SWISS-PROT:#CONTAM#P02533 Tax_Id=9606 Gene_Symbol=KRT14 Keratin type I cytoskeletal 14
115	P43652|AFAM_HUMAN	Afamin OS=Homo sapiens OX=9606 GN=AFM PE=1 SV=1
116	P04196|HRG_HUMAN	Histidine-rich glycoprotein OS=Homo sapiens OX=9606 GN=HRG PE=1 SV=1
117	Q96IY4|CBPB2_HUMAN	Carboxypeptidase B2 OS=Homo sapiens OX=9606 GN=CPB2 PE=1 SV=2

118	P05452|TETN_HUMAN	Tetranectin OS=Homo sapiens OX=9606 GN=CLEC3B PE=1 SV=3
119	Q4L235|ACSF4_HUMAN	Beta-alanine-activating enzyme OS=Homo sapiens OX=9606 GN=AASDH PE=1 SV=3
120	P35858|ALS_HUMAN	Insulin-like growth factor-binding protein complex acid labile subunit OS=Homo sapiens OX=9606 GN=IGFALS PE=1 SV=1
121	P51884|LUM_HUMAN	Lumican OS=Homo sapiens OX=9606 GN=LUM PE=1 SV=2
122	P07358|CO8B_HUMAN	Complement component C8 beta chain OS=Homo sapiens OX=9606 GN=C8B PE=1 SV=3
123	Q9ULW6|NP1L2_HUMAN	Nucleosome assembly protein 1-like 2 OS=Homo sapiens OX=9606 GN=NAP1L2 PE=1 SV=1
124	A2A2Z9|AN18B_HUMAN	Ankyrin repeat domain-containing protein 18B OS=Homo sapiens OX=9606 GN=ANKRD18B PE=1 SV=1
125	Q8IVF6|AN18A_HUMAN	Ankyrin repeat domain-containing protein 18A OS=Homo sapiens OX=9606 GN=ANKRD18A PE=2 SV=3
126	A0A0A0MS15|HV349_HUMAN	Immunoglobulin heavy variable 3-49 OS=Homo sapiens OX=9606 GN=IGHV3-49 PE=3 SV=1
127	A0A075B6Q5|HV364_HUMAN	Immunoglobulin heavy variable 3-64 OS=Homo sapiens OX=9606 GN=IGHV3-64 PE=3 SV=1
128	P13671|CO6_HUMAN	Complement component C6 OS=Homo sapiens OX=9606 GN=C6 PE=1 SV=3
129	A0A0J9YVY3|HV741_HUMAN	Immunoglobulin heavy variable 7-4-1 OS=Homo sapiens OX=9606 GN=IGHV7-4-1 PE=3 SV=1
130	P05155|IC1_HUMAN	Plasma protease C1 inhibitor OS=Homo sapiens OX=9606 GN=SERPING1 PE=1 SV=2
131	P36955|PEDF_HUMAN	Pigment epithelium-derived factor OS=Homo sapiens OX=9606 GN=SERPINF1 PE=1 SV=4
132	A0A0C4DH42|HV366_HUMAN	Immunoglobulin heavy variable 3-66 OS=Homo sapiens OX=9606 GN=IGHV3-66 PE=3 SV=1
133	A0A0B4J1V0|HV315_HUMAN	Immunoglobulin heavy variable 3-15 OS=Homo sapiens OX=9606 GN=IGHV3-15 PE=3 SV=1
134	P08185|CBG_HUMAN	Corticosteroid-binding globulin OS=Homo sapiens OX=9606 GN=SERPINA6 PE=1 SV=1
135	P00739|HPTR_HUMAN	Haptoglobin-related protein OS=Homo sapiens OX=9606 GN=HPR PE=2 SV=2
136	P01880|IGHD_HUMAN	Immunoglobulin heavy constant delta OS=Homo sapiens OX=9606 GN=IGHD PE=1 SV=3
137	A0A0B4J1Y9|HV372_HUMAN	Immunoglobulin heavy variable 3-72 OS=Homo sapiens OX=9606 GN=IGHV3-72 PE=3 SV=1
138	Q86XI8|ZSWM9_HUMAN	Uncharacterized protein ZSWIM9 OS=Homo sapiens OX=9606 GN=ZSWIM9 PE=1 SV=2
139	P53708|ITA8_HUMAN	Integrin alpha-8 OS=Homo sapiens OX=9606 GN=ITGA8 PE=1 SV=3
140	Q7Z745|MRO2B_HUMAN	Maestro heat-like repeat-containing protein family member 2B OS=Homo sapiens OX=9606 GN=MROH2B PE=2 SV=3
141	P02790|HEMO_HUMAN	Hemopexin OS=Homo sapiens OX=9606 GN=HPX PE=1 SV=2
142	Q8NE71|ABCF1_HUMAN	ATP-binding cassette sub-family F member 1 OS=Homo sapiens OX=9606 GN=ABCF1 PE=1 SV=2
143	Q14520|HABP2_HUMAN	Hyaluronan-binding protein 2 OS=Homo sapiens OX=9606 GN=HABP2 PE=1 SV=1
144	A0A0J9YX35|HV64D_HUMAN	Immunoglobulin heavy variable 3-64D OS=Homo sapiens OX=9606 GN=IGHV3-64D PE=3 SV=1
145	P01011|AACT_HUMAN	Alpha-1-antichymotrypsin OS=Homo sapiens OX=9606 GN=SERPINA3 PE=1 SV=2
146	P43251|BTD_HUMAN	Biotinidase OS=Homo sapiens OX=9606 GN=BTD PE=1 SV=2
147	P01780|HV307_HUMAN	Immunoglobulin heavy variable 3-7 OS=Homo sapiens OX=9606 GN=IGHV3-7 PE=1 SV=2
148	P04004|VTNC_HUMAN	Vitronectin OS=Homo sapiens OX=9606 GN=VTN PE=1 SV=1
149	A0A0C4DH38|HV551_HUMAN	Immunoglobulin heavy variable 5-51 OS=Homo sapiens OX=9606 GN=IGHV5-51 PE=3 SV=1
150	P25311|ZA2G_HUMAN	Zinc-alpha-2-glycoprotein OS=Homo sapiens OX=9606 GN=AZGP1 PE=1 SV=2

151	P01019|ANGT_HUMAN	Angiotensinogen OS=Homo sapiens OX=9606 GN=AGT PE=1 SV=1
152	P05543|THBG_HUMAN	Thyroxine-binding globulin OS=Homo sapiens OX=9606 GN=SERPINA7 PE=1 SV=2
153	P22352|GPX3_HUMAN	Glutathione peroxidase 3 OS=Homo sapiens OX=9606 GN=GPX3 PE=1 SV=2
154	P00747|PLMN_HUMAN	Plasminogen OS=Homo sapiens OX=9606 GN=PLG PE=1 SV=2
155	P06727|APOA4_HUMAN	Apolipoprotein A-IV OS=Homo sapiens OX=9606 GN=APOA4 PE=1 SV=3
156	P10909|CLUS_HUMAN	Clusterin OS=Homo sapiens OX=9606 GN=CLU PE=1 SV=1
157	A0A0C4DH69|KV109_HUMAN	Immunoglobulin kappa variable 1-9 OS=Homo sapiens OX=9606 GN=IGKV1-9 PE=3 SV=1
158	Q8NEN9|PDZD8_HUMAN	PDZ domain-containing protein 8 OS=Homo sapiens OX=9606 GN=PDZD8 PE=1 SV=1
159	P01024|CO3_HUMAN	Complement C3 OS=Homo sapiens OX=9606 GN=C3 PE=1 SV=2
160	P00742|FA10_HUMAN	Coagulation factor X OS=Homo sapiens OX=9606 GN=F10 PE=1 SV=2
161	P00751|CFAB_HUMAN	Complement factor B OS=Homo sapiens OX=9606 GN=CFB PE=1 SV=2
162	A0A0B4J2D9|KVD13_HUMAN	Immunoglobulin kappa variable 1D-13 OS=Homo sapiens OX=9606 GN=IGKV1D-13 PE=3 SV=1
163	P0DP09|KV113_HUMAN	Immunoglobulin kappa variable 1-13 OS=Homo sapiens OX=9606 GN=IGKV1-13 PE=3 SV=1
164	P02748|CO9_HUMAN	Complement component C9 OS=Homo sapiens OX=9606 GN=C9 PE=1 SV=2
165	P07357|CO8A_HUMAN	Complement component C8 alpha chain OS=Homo sapiens OX=9606 GN=C8A PE=1 SV=2
166	P03952|KLKB1_HUMAN	Plasma kallikrein OS=Homo sapiens OX=9606 GN=KLKB1 PE=1 SV=1
167	P02652|APOA2_HUMAN	Apolipoprotein A-II OS=Homo sapiens OX=9606 GN=APOA2 PE=1 SV=1
168	P04217|A1BG_HUMAN	Alpha-1B-glycoprotein OS=Homo sapiens OX=9606 GN=A1BG PE=1 SV=4
169	P00748|FA12_HUMAN	Coagulation factor XII OS=Homo sapiens OX=9606 GN=F12 PE=1 SV=3
170	Q15848|ADIPO_HUMAN	Adiponectin OS=Homo sapiens OX=9606 GN=ADIPOQ PE=1 SV=1
171	P00740|FA9_HUMAN	Coagulation factor IX OS=Homo sapiens OX=9606 GN=F9 PE=1 SV=2
172	O60307|MAST3_HUMAN	Microtubule-associated serine/threonine-protein kinase 3 OS=Homo sapiens OX=9606 GN=MAST3 PE=1 SV=2
173	P10643|CO7_HUMAN	Complement component C7 OS=Homo sapiens OX=9606 GN=C7 PE=1 SV=2
174	P05160|F13B_HUMAN	Coagulation factor XIII B chain OS=Homo sapiens OX=9606 GN=F13B PE=1 SV=3
175	P19823|ITIH2_HUMAN	Inter-alpha-trypsin inhibitor heavy chain H2 OS=Homo sapiens OX=9606 GN=ITIH2 PE=1 SV=2
176	P01602|KV105_HUMAN	Immunoglobulin kappa variable 1-5 OS=Homo sapiens OX=9606 GN=IGKV1-5 PE=1 SV=2
177	P01009|A1AT_HUMAN	Alpha-1-antitrypsin OS=Homo sapiens OX=9606 GN=SERPINA1 PE=1 SV=3
178	Q9HCJ0|TNR6C_HUMAN	Trinucleotide repeat-containing gene 6C protein OS=Homo sapiens OX=9606 GN=TNRC6C PE=1 SV=3
179	A0A0J9YXX1|HV5X1_HUMAN	Immunoglobulin heavy variable 5-10-1 OS=Homo sapiens OX=9606 GN=IGHV5-10-1 PE=3 SV=1
180	P07360|CO8G_HUMAN	Complement component C8 gamma chain OS=Homo sapiens OX=9606 GN=C8G PE=1 SV=3
181	P02787|TRFE_HUMAN	Serotransferrin OS=Homo sapiens OX=9606 GN=TF PE=1 SV=3
182	Q96PD5|PGRP2_HUMAN	N-acetylmuramoyl-L-alanine amidase OS=Homo sapiens OX=9606 GN=PGLYRP2 PE=1 SV=1
183	Q9NZP8|C1RL_HUMAN	Complement C1r subcomponent-like protein OS=Homo sapiens OX=9606 GN=C1RL PE=1 SV=2

184	P01714|LV319_HUMAN	Immunoglobulin lambda variable 3-19 OS=Homo sapiens OX=9606 GN=IGLV3-19 PE=1 SV=2
185	B9A064|IGLL5_HUMAN	Immunoglobulin lambda-like polypeptide 5 OS=Homo sapiens OX=9606 GN=IGLL5 PE=2 SV=2
186	P01762|HV311_HUMAN	Immunoglobulin heavy variable 3-11 OS=Homo sapiens OX=9606 GN=IGHV3-11 PE=1 SV=2
187	Q9NYR9|KBRS2_HUMAN	NF-kappa-B inhibitor-interacting Ras-like protein 2 OS=Homo sapiens OX=9606 GN=NKIRAS2 PE=1 SV=1
188	P00450|CERU_HUMAN	Ceruloplasmin OS=Homo sapiens OX=9606 GN=CP PE=1 SV=1
189	P01718|LV327_HUMAN	Immunoglobulin lambda variable 3-27 OS=Homo sapiens OX=9606 GN=IGLV3-27 PE=1 SV=2
190	P02647|APOA1_HUMAN	Apolipoprotein A-I OS=Homo sapiens OX=9606 GN=APOA1 PE=1 SV=1
191	P04211|LV743_HUMAN	Immunoglobulin lambda variable 7-43 OS=Homo sapiens OX=9606 GN=IGLV7-43 PE=3 SV=2
192	A0A075B6I9|LV746_HUMAN	Immunoglobulin lambda variable 7-46 OS=Homo sapiens OX=9606 GN=IGLV7-46 PE=3 SV=4
193	P0C7P1|RBY1D_HUMAN	RNA-binding motif protein Y chromosome family 1 member D OS=Homo sapiens OX=9606 GN=RBMY1D PE=2 SV=1
194	P0DJD3|RBY1A_HUMAN	RNA-binding motif protein Y chromosome family 1 member A1 OS=Homo sapiens OX=9606 GN=RBMY1A1 PE=1 SV=1
195	A6NEQ0|RBY1E_HUMAN	RNA-binding motif protein Y chromosome family 1 member E OS=Homo sapiens OX=9606 GN=RBMY1E PE=2 SV=1
196	P0DJD4|RBY1C_HUMAN	RNA-binding motif protein Y chromosome family 1 member C OS=Homo sapiens OX=9606 GN=RBMY1C PE=1 SV=1
197	A6NDE4|RBY1B_HUMAN	RNA-binding motif protein Y chromosome family 1 member B OS=Homo sapiens OX=9606 GN=RBMY1B PE=2 SV=2
198	Q15415|RBY1F_HUMAN	RNA-binding motif protein Y chromosome family 1 member F/J OS=Homo sapiens OX=9606 GN=RBMY1F PE=1 SV=2
199	P0C0L4|CO4A_HUMAN	Complement C4-A OS=Homo sapiens OX=9606 GN=C4A PE=1 SV=2
200	A0A0A0MRZ8|KVD11_HUMAN	Immunoglobulin kappa variable 3D-11 OS=Homo sapiens OX=9606 GN=IGKV3D-11 PE=3 SV=6
201	P04433|KV311_HUMAN	Immunoglobulin kappa variable 3-11 OS=Homo sapiens OX=9606 GN=IGKV3-11 PE=1 SV=1
202	P21359|NF1_HUMAN	Neurofibromin OS=Homo sapiens OX=9606 GN=NF1 PE=1 SV=2
203	Q9Y625|GPC6_HUMAN	Glypican-6 OS=Homo sapiens OX=9606 GN=GPC6 PE=1 SV=1
204	Q16610|ECM1_HUMAN	Extracellular matrix protein 1 OS=Homo sapiens OX=9606 GN=ECM1 PE=1 SV=2
205	P02753|RET4_HUMAN	Retinol-binding protein 4 OS=Homo sapiens OX=9606 GN=RBP4 PE=1 SV=3
206	P01593|KVD33_HUMAN	Immunoglobulin kappa variable 1D-33 OS=Homo sapiens OX=9606 GN=IGKV1D-33 PE=1 SV=2
207	P01594|KV133_HUMAN	Immunoglobulin kappa variable 1-33 OS=Homo sapiens OX=9606 GN=IGKV1-33 PE=1 SV=2
208	P00734|THRB_HUMAN	Prothrombin OS=Homo sapiens OX=9606 GN=F2 PE=1 SV=2
209	A0A0B4J1U7|HV601_HUMAN	Immunoglobulin heavy variable 6-1 OS=Homo sapiens OX=9606 GN=IGHV6-1 PE=3 SV=1
210	A0A075B6I0|LV861_HUMAN	Immunoglobulin lambda variable 8-61 OS=Homo sapiens OX=9606 GN=IGLV8-61 PE=3 SV=7
211	Q9UK55|ZPI_HUMAN	Protein Z-dependent protease inhibitor OS=Homo sapiens OX=9606 GN=SERPINA10 PE=1 SV=1
212	#CONTAM#P02768-1	SWISS-PROT:#CONTAM#P02768-1 Tax_Id=9606 Gene_Symbol=ALB Isoform 1 of Serum albumin precursor
213	P02768|ALBU_HUMAN	Serum albumin OS=Homo sapiens OX=9606 GN=ALB PE=1 SV=2
214	P04278|SHBG_HUMAN	Sex hormone-binding globulin OS=Homo sapiens OX=9606 GN=SHBG PE=1 SV=2
215	P0C0L5|CO4B_HUMAN	Complement C4-B OS=Homo sapiens OX=9606 GN=C4B PE=1 SV=2
216	P01031|CO5_HUMAN	Complement C5 OS=Homo sapiens OX=9606 GN=C5 PE=1 SV=4

217	P01703|LV140_HUMAN	Immunoglobulin lambda variable 1-40 OS=Homo sapiens OX=9606 GN=IGLV1-40 PE=1 SV=2
218	A0A0C4DH73|KV112_HUMAN	Immunoglobulin kappa variable 1-12 OS=Homo sapiens OX=9606 GN=IGKV1-12 PE=3 SV=1
219	P01611|KVD12_HUMAN	Immunoglobulin kappa variable 1D-12 OS=Homo sapiens OX=9606 GN=IGKV1D-12 PE=1 SV=2
220	P08697|A2AP_HUMAN	Alpha-2-antiplasmin OS=Homo sapiens OX=9606 GN=SERPINF2 PE=1 SV=3
221	P01619|KV320_HUMAN	Immunoglobulin kappa variable 3-20 OS=Homo sapiens OX=9606 GN=IGKV3-20 PE=1 SV=2
222	P01860|IGHG3_HUMAN	Immunoglobulin heavy constant gamma 3 OS=Homo sapiens OX=9606 GN=IGHG3 PE=1 SV=2
223	P55058|PLTP_HUMAN	Phospholipid transfer protein OS=Homo sapiens OX=9606 GN=PLTP PE=1 SV=1
224	P22792|CPN2_HUMAN	Carboxypeptidase N subunit 2 OS=Homo sapiens OX=9606 GN=CPN2 PE=1 SV=3
225	P00738|HPT_HUMAN	Haptoglobin OS=Homo sapiens OX=9606 GN=HP PE=1 SV=1
226	P32004|L1CAM_HUMAN	Neural cell adhesion molecule L1 OS=Homo sapiens OX=9606 GN=L1CAM PE=1 SV=2
227	Q13790|APOF_HUMAN	Apolipoprotein F OS=Homo sapiens OX=9606 GN=APOF PE=1 SV=2
228	P06396|GELS_HUMAN	Gelsolin OS=Homo sapiens OX=9606 GN=GSN PE=1 SV=1
229	P01859|IGHG2_HUMAN	Immunoglobulin heavy constant gamma 2 OS=Homo sapiens OX=9606 GN=IGHG2 PE=1 SV=2
230	O75882|ATRN_HUMAN	Attractin OS=Homo sapiens OX=9606 GN=ATRN PE=1 SV=2
231	O00410|IPO5_HUMAN	Importin-5 OS=Homo sapiens OX=9606 GN=IPO5 PE=1 SV=4
232	Q14624|ITIH4_HUMAN	Inter-alpha-trypsin inhibitor heavy chain H4 OS=Homo sapiens OX=9606 GN=ITIH4 PE=1 SV=4
233	P05546|HEP2_HUMAN	Heparin cofactor 2 OS=Homo sapiens OX=9606 GN=SERPIND1 PE=1 SV=3
234	P01857|IGHG1_HUMAN	Immunoglobulin heavy constant gamma 1 OS=Homo sapiens OX=9606 GN=IGHG1 PE=1 SV=1
235	O95445|APOM_HUMAN	Apolipoprotein M OS=Homo sapiens OX=9606 GN=APOM PE=1 SV=2
236	P20701|ITAL_HUMAN	Integrin alpha-L OS=Homo sapiens OX=9606 GN=ITGAL PE=1 SV=3
237	P27169|PON1_HUMAN	Serum paraoxonase/arylesterase 1 OS=Homo sapiens OX=9606 GN=PON1 PE=1 SV=3
238	P01877|IGHA2_HUMAN	Immunoglobulin heavy constant alpha 2 OS=Homo sapiens OX=9606 GN=IGHA2 PE=1 SV=4
239	Q99684|GFI1_HUMAN	Zinc finger protein Gfi-1 OS=Homo sapiens OX=9606 GN=GFI1 PE=1 SV=2
240	A0A0C4DH72|KV106_HUMAN	Immunoglobulin kappa variable 1-6 OS=Homo sapiens OX=9606 GN=IGKV1-6 PE=3 SV=1
241	Q9UGM5|FETUB_HUMAN	Fetuin-B OS=Homo sapiens OX=9606 GN=FETUB PE=1 SV=2
242	#CONTAM#P00761	SWISS-PROT:#CONTAM#P00761|TRYP_PIG Trypsin - Sus scrofa (Pig).
243	P01615|KVD28_HUMAN	Immunoglobulin kappa variable 2D-28 OS=Homo sapiens OX=9606 GN=IGKV2D-28 PE=1 SV=2
244	A0A075B6P5|KV228_HUMAN	Immunoglobulin kappa variable 2-28 OS=Homo sapiens OX=9606 GN=IGKV2-28 PE=3 SV=1
245	Q99988|GDF15_HUMAN	Growth/differentiation factor 15 OS=Homo sapiens OX=9606 GN=GDF15 PE=1 SV=3
246	A0A0B4J1X5|HV374_HUMAN	Immunoglobulin heavy variable 3-74 OS=Homo sapiens OX=9606 GN=IGHV3-74 PE=3 SV=1
247	P01709|LV208_HUMAN	Immunoglobulin lambda variable 2-8 OS=Homo sapiens OX=9606 GN=IGLV2-8 PE=1 SV=2
248	P01721|LV657_HUMAN	Immunoglobulin lambda variable 6-57 OS=Homo sapiens OX=9606 GN=IGLV6-57 PE=1 SV=2
249	Q5VT06|CE350_HUMAN	Centrosome-associated protein 350 OS=Homo sapiens OX=9606 GN=CEP350 PE=1 SV=1

250	A0A0C4DH68|KV224_HUMAN	Immunoglobulin kappa variable 2-24 OS=Homo sapiens OX=9606 GN=IGKV2-24 PE=3 SV=1
251	P01834|IGKC_HUMAN	Immunoglobulin kappa constant OS=Homo sapiens OX=9606 GN=IGKC PE=1 SV=2
252	P02766|TTHY_HUMAN	Transthyretin OS=Homo sapiens OX=9606 GN=TTR PE=1 SV=1
253	P23142|FBLN1_HUMAN	Fibulin-1 OS=Homo sapiens OX=9606 GN=FBLN1 PE=1 SV=4
254	A0A075B6S6|KVD30_HUMAN	Immunoglobulin kappa variable 2D-30 OS=Homo sapiens OX=9606 GN=IGKV2D-30 PE=3 SV=1
255	P06310|KV230_HUMAN	Immunoglobulin kappa variable 2-30 OS=Homo sapiens OX=9606 GN=IGKV2-30 PE=3 SV=2
256	P05090|APOD_HUMAN	Apolipoprotein D OS=Homo sapiens OX=9606 GN=APOD PE=1 SV=1
257	P02760|AMBP_HUMAN	Protein AMBP OS=Homo sapiens OX=9606 GN=AMBP PE=1 SV=1
258	P49908|SEPP1_HUMAN	Selenoprotein P OS=Homo sapiens OX=9606 GN=SELENOP PE=1 SV=3
259	P15814|IGLL1_HUMAN	Immunoglobulin lambda-like polypeptide 1 OS=Homo sapiens OX=9606 GN=IGLL1 PE=1 SV=1
260	P02671|FIBA_HUMAN	Fibrinogen alpha chain OS=Homo sapiens OX=9606 GN=FGA PE=1 SV=2
261	P01861|IGHG4_HUMAN	Immunoglobulin heavy constant gamma 4 OS=Homo sapiens OX=9606 GN=IGHG4 PE=1 SV=1
262	P01876|IGHA1_HUMAN	Immunoglobulin heavy constant alpha 1 OS=Homo sapiens OX=9606 GN=IGHA1 PE=1 SV=2
263	P15169|CBPN_HUMAN	Carboxypeptidase N catalytic chain OS=Homo sapiens OX=9606 GN=CPN1 PE=1 SV=1
264	P01700|LV147_HUMAN	Immunoglobulin lambda variable 1-47 OS=Homo sapiens OX=9606 GN=IGLV1-47 PE=1 SV=2
265	P22891|PROZ_HUMAN	Vitamin K-dependent protein Z OS=Homo sapiens OX=9606 GN=PROZ PE=1 SV=2
266	P17936|IBP3_HUMAN	Insulin-like growth factor-binding protein 3 OS=Homo sapiens OX=9606 GN=IGFBP3 PE=1 SV=2
267	O14791|APOL1_HUMAN	Apolipoprotein L1 OS=Homo sapiens OX=9606 GN=APOL1 PE=1 SV=5
268	P06331|HV434_HUMAN	Immunoglobulin heavy variable 4-34 OS=Homo sapiens OX=9606 GN=IGHV4-34 PE=1 SV=2
269	P00439|PH4H_HUMAN	Phenylalanine-4-hydroxylase OS=Homo sapiens OX=9606 GN=PAH PE=1 SV=1
270	P07225|PROS_HUMAN	Vitamin K-dependent protein S OS=Homo sapiens OX=9606 GN=PROS1 PE=1 SV=1
271	#CONTAM#Q1RMN8	TREMBL:#CONTAM#Q1RMN8 (Bos taurus) Similar to Immunoglobulin lambda-like polypeptide 1
272	P01705|LV223_HUMAN	Immunoglobulin lambda variable 2-23 OS=Homo sapiens OX=9606 GN=IGLV2-23 PE=1 SV=2
273	P19827|ITIH1_HUMAN	Inter-alpha-trypsin inhibitor heavy chain H1 OS=Homo sapiens OX=9606 GN=ITIH1 PE=1 SV=3
274	A0A0C4DH32|HV320_HUMAN	Immunoglobulin heavy variable 3-20 OS=Homo sapiens OX=9606 GN=IGHV3-20 PE=3 SV=2
275	P80108|PHLD_HUMAN	Phosphatidylinositol-glycan-specific phospholipase D OS=Homo sapiens OX=9606 GN=GPLD1 PE=1 SV=3
276	Q04756|HGFA_HUMAN	Hepatocyte growth factor activator OS=Homo sapiens OX=9606 GN=HGFAC PE=1 SV=1
277	P04070|PROC_HUMAN	Vitamin K-dependent protein C OS=Homo sapiens OX=9606 GN=PROC PE=1 SV=1
278	P01704|LV214_HUMAN	Immunoglobulin lambda variable 2-14 OS=Homo sapiens OX=9606 GN=IGLV2-14 PE=1 SV=2
279	Q14CM0|FRPD4_HUMAN	FERM and PDZ domain-containing protein 4 OS=Homo sapiens OX=9606 GN=FRMPD4 PE=1 SV=1
280	A0A0B4J1V1|HV321_HUMAN	Immunoglobulin heavy variable 3-21 OS=Homo sapiens OX=9606 GN=IGHV3-21 PE=1 SV=1
281	Q8NDX1|PSD4_HUMAN	PH and SEC7 domain-containing protein 4 OS=Homo sapiens OX=9606 GN=PSD4 PE=1 SV=2
282	A0A075B6R2|HV404_HUMAN	Immunoglobulin heavy variable 4-4 OS=Homo sapiens OX=9606 GN=IGHV4-4 PE=3 SV=2

283	Q9NZ63|TLS1_HUMAN	Telomere length and silencing protein 1 homolog OS=Homo sapiens OX=9606 GN=C9orf78 PE=1 SV=1
284	P35542|SAA4_HUMAN	Serum amyloid A-4 protein OS=Homo sapiens OX=9606 GN=SAA4 PE=1 SV=2
285	P0DJI8|SAA1_HUMAN	Serum amyloid A-1 protein OS=Homo sapiens OX=9606 GN=SAA1 PE=1 SV=1
286	Q05397|FAK1_HUMAN	Focal adhesion kinase 1 OS=Homo sapiens OX=9606 GN=PTK2 PE=1 SV=2
287	P80748|LV321_HUMAN	Immunoglobulin lambda variable 3-21 OS=Homo sapiens OX=9606 GN=IGLV3-21 PE=1 SV=2
288	P02675|FIBB_HUMAN	Fibrinogen beta chain OS=Homo sapiens OX=9606 GN=FGB PE=1 SV=2
289	A0A0C4DH24|KV621_HUMAN	Immunoglobulin kappa variable 6-21 OS=Homo sapiens OX=9606 GN=IGKV6-21 PE=3 SV=1
290	P0DOY3|IGLC3_HUMAN	Immunoglobulin lambda constant 3 OS=Homo sapiens OX=9606 GN=IGLC3 PE=1 SV=1
291	P0DOY2|IGLC2_HUMAN	Immunoglobulin lambda constant 2 OS=Homo sapiens OX=9606 GN=IGLC2 PE=1 SV=1
292	Q06033|ITIH3_HUMAN	Inter-alpha-trypsin inhibitor heavy chain H3 OS=Homo sapiens OX=9606 GN=ITIH3 PE=1 SV=2
293	P02655|APOC2_HUMAN	Apolipoprotein C-II OS=Homo sapiens OX=9606 GN=APOC2 PE=1 SV=1
294	P02679|FIBG_HUMAN	Fibrinogen gamma chain OS=Homo sapiens OX=9606 GN=FGG PE=1 SV=3
295	P02649|APOE_HUMAN	Apolipoprotein E OS=Homo sapiens OX=9606 GN=APOE PE=1 SV=1
296	P01023|A2MG_HUMAN	Alpha-2-macroglobulin OS=Homo sapiens OX=9606 GN=A2M PE=1 SV=3
297	#CONTAM#ENSEMBL:ENSBTAP00000024466	(Bos taurus) 44 kDa protein
298	P02656|APOC3_HUMAN	Apolipoprotein C-III OS=Homo sapiens OX=9606 GN=APOC3 PE=1 SV=1
299	P01871|IGHM_HUMAN	Immunoglobulin heavy constant mu OS=Homo sapiens OX=9606 GN=IGHM PE=1 SV=4
300	P01766|HV313_HUMAN	Immunoglobulin heavy variable 3-13 OS=Homo sapiens OX=9606 GN=IGHV3-13 PE=1 SV=2
301	P09871|C1S_HUMAN	Complement C1s subcomponent OS=Homo sapiens OX=9606 GN=C1S PE=1 SV=1
302	P02743|SAMP_HUMAN	Serum amyloid P-component OS=Homo sapiens OX=9606 GN=APCS PE=1 SV=2
303	P69905|HBA_HUMAN	Hemoglobin subunit alpha OS=Homo sapiens OX=9606 GN=HBA1 PE=1 SV=2
